# Creasing in microscale, soft static friction

**DOI:** 10.1038/s41467-023-38091-7

**Published:** 2023-04-24

**Authors:** Justin D. Glover, Xingwei Yang, Rong Long, Jonathan T. Pham

**Affiliations:** 1 Department of Chemical and Materials Engineering, University of Kentucky, Lexington, KY 40506 USA; 2 Department of Mechanical Engineering, University of Colorado Boulder, Boulder, CO 80309 USA; 3 Department of Chemical and Environmental Engineering, University of Cincinnati, Cincinnati, OH 45221 USA

**Keywords:** Soft materials, Mechanical engineering

## Abstract

Utilizing colloidal probe, lateral force microscopy and simultaneous confocal microscopy, combined with finite element analysis, we investigate how a microparticle starts moving laterally on a soft, adhesive surface. We find that the surface can form a self-contacting crease at the leading front, which results from a buildup of compressive stress. Experimentally, creases are observed on substrates that exhibit either high or low adhesion when measured in the normal direction, motivating the use of simulations to consider the role of adhesion energy and interfacial strength. Our simulations illustrate that the interfacial strength plays a dominating role in the nucleation of a crease. After the crease forms, it progresses through the contact zone in a Schallamach wave-like fashion. Interestingly, our results suggest that this Schallamach wave-like motion is facilitated by free slip at the adhesive, self-contacting interface within the crease.

## Abstract (web summary)

Soft friction remains elusive due to the complication at microscales where the elastic forces are comparable to capillarity and adhesion. Glover et al. show that a moving microparticle can induce a cease at the leading front of the underlying soft surface as a result of a build-up of compressive stress.

## Introduction

Understanding static friction of soft materials is important for a range of applications, from bioinspired adhesives^1–3^ and car tires^4^ to damage of articular cartilage^5^. Although studies on friction started half a millennium ago by da Vinci^6^, fundamental challenges persist for soft adhesive interfaces^7,8^. In its most basic form, consider an elastomeric block attached to a spring, which is pulled on a rigid surface. The block remains stationary until the spring force overcomes a threshold, at which point the block moves relative to the surface; this static threshold is related to a number of parameters, like the contact area^1,9–11^. For macroscopic surfaces, the true contact area is related to microscale roughness^9,12^, where microscopic contact points at the interface often control how the two surfaces interact^9,13,14^. However, for very soft materials that have a modulus of a few kPa, probed on microscopic scales, elastic forces approach those of capillarity and adhesion, leading to unique physics that are still not fully understood (e.g. elastocapillarity). This leads to a gap in our knowledge about how a single microscopic point, like a spherical microparticle, starts to move on a soft adhesive surface.

In studies of soft tribology and adhesion, a spherical probe is commonly used as a model geometry^9,14–20^. When a sphere is pulled laterally on an elastomer, adhesive interactions lead to the static friction threshold^21^. To overcome this threshold, Schallamach waves can emerge to enable relative motion. First reported in 1971, Schallamach waves describe a cascade of detachment and reattachment events that propagate through the contact zone^22,23^. These waves form when a buckling event occurs at the front of the sphere, which then reattaches to the sphere and creates a pocket of air. This air gap then moves through the contact zone, like a ruck moving through a rug^24^, without significant interfacial slippage between the sphere and the substrate. This air gap can also vanish under the contact zone, allowing the sphere and surface to make new contact^25^. Schallamach waves are the most reported instance of surface buckling behavior in soft, tribological contacts; however, most experiments have focused on macroscale spheres^23,26–31^. As we will show here on the microscale, the ruck through a rug mechanism is no longer an air pocket, but a self-contacting, non-damaging crease.

Creases are surface instabilities resulting from a state of compressive strain. When the surface of a material is compressed, for example through direct compression or constrained swelling, wrinkles and creases develop. Wrinkles are sinusoidal undulations, whereas creases are localized, self-contacting, fold-like geometries. These instabilities can create microscale surface features, which have been used to tune adhesion, pattern surfaces, and control biological cells^32,33^. While the onset, growth, and recovery of creases have been and still are under investigation, the focus has been on soft substrates under uniform compression^34–36^. On the other hand, soft friction can also generate compression near the contact zone, but in a localized, asymmetric manner. This generates a question of how creases form in a tribological setting and their potential coupling to friction.

By combining confocal microscopy, (colloidal probe) lateral force microscopy, and numerical simulations, we study how a stiff microparticle starts moving laterally on a very soft adhesive surface. A self-contacting crease forms at the front of the microparticle, demonstrating a link between tribology and creasing. Our experiments illustrate that crease formation occurs in situations of either low or high adhesion, measured normal to the surface. This motivates simulations to consider the role of adhesion energy versus interfacial strength in the emergence of creases. Simulations demonstrate that interfacial strength, i.e., the highest stress that can be sustained by the microparticle–soft surface interface, is the governing factor to initiate creasing. For creases to grow and fully progress through the contact zone, as observed in our experiments, the self-contacting crease interface should be adhesive, while still allowing for slip. Hence, the conditions for crease formation and progression include (1) little to no slippage between the microsphere and the substrate and (2) sufficient normal adhesion but allowable slip within the crease itself—that is, an adhesive yet self-slippery crease interface. Additionally, these creases re-open on the trailing edge without leaving a permanent scar, demonstrating a no-wear tribological situation.

## Results

### Experimental approach and initial observations

**Fig. 1:**
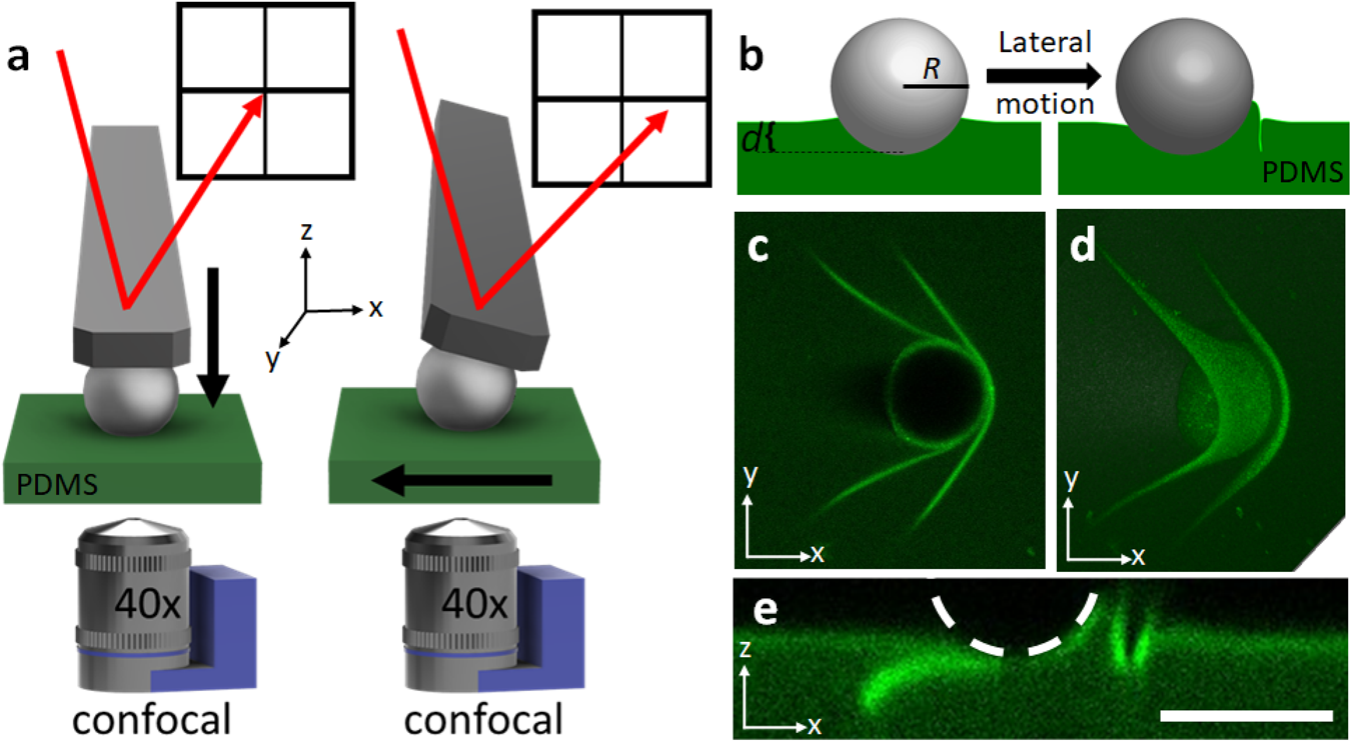
Experimental observations of a microparticle starting to be pulled laterally on a soft elastomer. **a** Schematic of the experimental setup showing the combined AFM cantilever and confocal microscope used to simultaneously image the contact and measure the lateral force. **b** Schematic of crease formation, also showing the experimental variables of depth, $d,$ and radius, $R$. **c** A *x*–*y* confocal image of a microsphere ($R=8.5$ µm) with two creases; one crease is under and the other is in front of the microsphere. **d** 3D image of the same microsphere viewed from below. Note that the particle is stopped for high-resolution 3D images, rather than a single 2D slice. **e** A *x*–*z* cross-sectional image showing two creases. Scale bar: 20 ${{{{{\rm{\mu }}}}}}{{{{{\rm{m}}}}}}$.

For our experiments, we use a colloidal probe (microsphere) on an atomic force microscope (AFM), combined with a high-precision stage, to apply lateral displacements and measure lateral forces on a soft, polydimethylsiloxane (PDMS) elastomer (Fig. 1a). To image the substrate, a confocal microscope with a piezo-driven objective is used for fast, in-situ imaging of *x*–*z* cross-sections. For the PDMS substrate, Sylgard 184 is mixed at a 60:1 base:crosslinker ratio and spin-coated on a glass slide to a thickness of ~90 µm. Prior to spin-coating, a fluorescent monomer is added that binds to the polymer network, enabling fluorescent imaging by confocal microscopy^37–39^. Upon curing, a crosslinked film is obtained with Young’s modulus of ~4 kPa^37,40,41^. For the colloidal probe, an 8.5 µm radius (*R*) glass sphere is attached to an AFM cantilever.

In a typical experiment, the sphere is brought into contact with the surface to a desired depth, $d$, and held until a force plateau, which is between 15 and 45 s. The substrate is subsequently translated laterally at a pre-defined velocity, $v$, while simultaneously imaging the contact (Fig. 1a). To measure the lateral force, ${F}_{{{{{{\rm{L}}}}}}}$, a laser bounces off the cantilever into a detector to quantify the cantilever deflection. As the PDMS is pulled laterally relative to the stationary sphere, the cantilever deflects and changes the laser position (Fig. 1a). To investigate the effect of indentation depth, the relative depth is controlled to a range of $d/R$ ~ 0.03–0.9. The upper limit is chosen such that the substrate meniscus remains at or below the center of the sphere prior to translation, while the lower limit is set naturally by adhesion, which slightly pulls the sphere into the substrate. Since rate has been shown to affect friction behavior, three velocities are chosen over a few decades: *v* = 10, 1, and 0.1 µm s^−1^ ^18,42^.

**Fig. 2:**
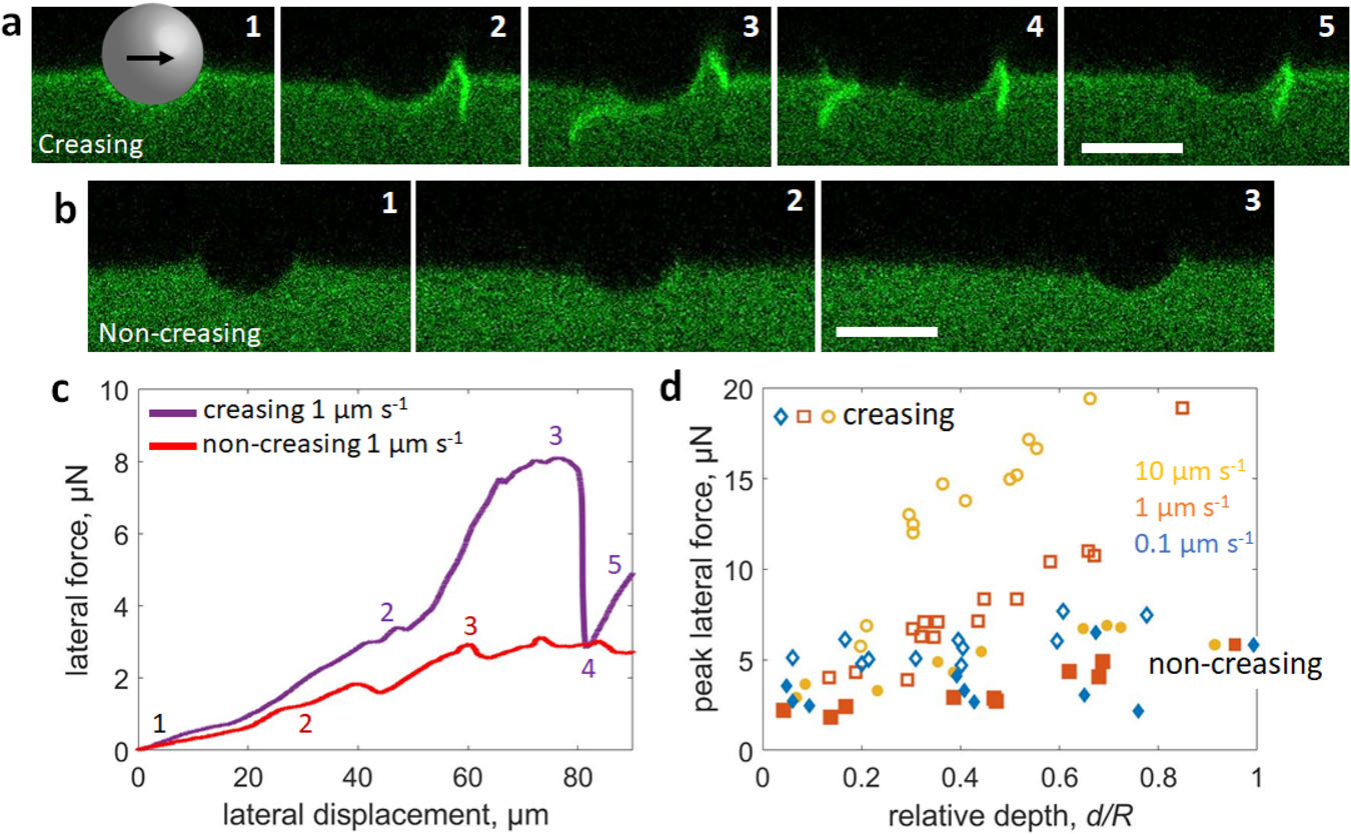
Creasing and non-creasing cases and the corresponding lateral forces. **a** Confocal *x*–*z* images of a creasing sample moving at $v$ = 1 µm s^−1^ with $d/R$= 0.4. **a**1 The microsphere is in contact with the surface prior to the start of motion. **a**2 Formation of a first crease. **a**3 The moment before the first crease is about to release on the trailing edge. **a**4 First image frame after the first crease releases and the force drops. **a**5 Second frame after the first crease releases, showing an uncreased state. **b** Confocal *x*–*z* images of a non-creasing sample moving at $v$ = 1 µm s^−1^ with $d/R$= 0.4. **b**1 The microsphere in contact with the surface prior to the start of motion. **b**2 The contact shape is about midway to the peak lateral force (${F}_{{{{{{\rm{peak}}}}}}}$). **b**3 The contact shape once the lateral force ${F}_{{{{{{\rm{L}}}}}}}$ has reached an approximate force plateau. **c** Lateral force (${F}_{{{{{{\rm{L}}}}}}}$) vs lateral displacement curves for creasing and non-creasing samples. The numbered points correlate with the confocal images in parts (**a**) and (**b**). **d** The peak lateral force $({F}_{{{{{{\rm{peak}}}}}}})$ for creasing and non-creasing samples as a function of $d/R$ and $v$. Scale bars: 20 ${{{{{\rm{\mu }}}}}}{{{{{\rm{m}}}}}}$.

Upon translation, our initial observations showed that a crease emerges in the PDMS substrate at the front of the microsphere (Fig. 1b). As the substrate continues to translate, a second crease can also form before the first crease is released on the trailing edge. Example confocal images of creases are shown in Fig. 1c–e from different viewing directions, depicting a full picture of the 3D crease geometry. As illustrated in a bottom-view *x*–*y* image (Fig. 1c), the crease extends outside the contact zone of the microsphere. A 3D bottom view (Fig. 1d) shows that the first crease stretches underneath the microsphere, while a second crease forms at the leading edge. 3D in this case means that an image stack is displayed, rather than a single 2D slice. A cross-sectional *x*–*z* image through the microsphere centerline (Fig. 1e) clearly illustrates the crease geometry. A movie of the entire process of crease formation during translation is presented in the Supplementary Information (Supplementary Movie 1), with snapshots of important events provided in Fig. 2a.

It is important to note that crease formation is related to the details of the substrate preparation methods. In the case of Fig. 1, the substrate is left in ambient, lit conditions for 4 days. However, when samples are tested directly after curing, creases are not observed. A movie of the non-creasing case is presented in the Supplementary Information (Supplementary Movie 2), with snapshots of important events provided in Fig. 2b. Note that we will use the term light-aging to describe the amount of time the free PDMS surface is left in lit conditions, prior to colloidal probe experiments. To confirm that light-aging is the reason for crease formation, we perform several control experiments to test small changes in Sylgard 184 mixing ratio (e.g. modulus), particle size, dwell times, sample thickness, and extended curing times (see Supplementary Note 1). This difference in crease behavior after light-aging may be the result of changes in crosslinking over time^43^, combined with UV exposure altering the surface^44,45^. Our results on creasing and non-creasing are reproducible, which offers an approach to investigate the physical parameters that govern creasing and the corresponding lateral force. Additionally, within our experimental capabilities, we find that the dye itself does not affect the crease formation or force behavior (Supplementary Fig. 1).

To quantitatively compare the lateral resistance of the two cases, we use freshly prepared samples and those aged for 4 days in lit ambient conditions. In Fig. 2c, an example lateral force (${F}_{{{{{{\rm{L}}}}}}}$) versus lateral displacement curve is plotted for creasing (purple curve) and non-creasing (red curve) cases ($d/R=0.4$ and *v* = 1 µm s^−1^). With the benefit of simultaneous imaging, ${F}_{{{{{{\rm{L}}}}}}}$ is correlated to confocal images to gain insight into the effect of creasing on the lateral force. The numbered spots in Fig. 2c correspond to the snapshots in Fig. 2a and b. For example, at spot 1 on Fig. 2c, the microsphere is in contact but at rest on the surface (Fig. 2a1 and b1). Upon translating the substrate for the creasing case, ${F}_{{{{{{\rm{L}}}}}}}$ initially increases while the microparticle remains attached to the substrate. At spot 2, a crease emerges, leading to a small reduction in ${F}_{{{{{{\rm{L}}}}}}}$; this reduction in ${F}_{{{{{{\rm{L}}}}}}}$ is afforded by increased compliance from the creased surface. Once the crease is in full self-contact, ${F}_{{{{{{\rm{L}}}}}}}$ continues to climb and approaches a peak at spot 3. At this point, the first crease is under the microparticle (Fig. 2a3). Upon translating beyond the peak lateral force (defined as ${F}_{{{{{{\rm{peak}}}}}}}$), the first crease detaches on the trailing edge (Fig. 2a4) and ${F}_{{{{{{\rm{L}}}}}}}$ drops precipitously to spot 4, returning the surface to an uncreased state (Fig. 2a5). For the non-creasing case, ${F}_{{{{{{\rm{L}}}}}}}$ initially increases, like for the creasing case. However, a clear difference is observed between the creasing and non-creasing force plots. At spot 2 (Fig. 2b2), the height of the PDMS contact line at the back of the microparticle lowers, while no change is evident at the front. After reaching ${F}_{{{{{{\rm{peak}}}}}}}$, the force is in an approximate plateau region and the most obvious change in deformation geometry is the lower meniscus height at the trailing edge. Some minor stick-slip is observed (Supplementary Movie 2), which relates to fluctuations in ${F}_{{{{{{\rm{L}}}}}}}$.

As demonstrated by Fig. 2c, ${F}_{{{{{{\rm{peak}}}}}}}$ is significantly higher for creasing compared to the non-creasing case. To consider whether different testing parameters play a role, ${F}_{{{{{{\rm{peak}}}}}}}$ is plotted as a function of $d/R$ and *v* for both cases (Fig. 2d). In general, increasing both *d*/*R* and *v* increase *F*_peak_ within each case of creasing or non-creasing. The increase in *F*_peak_ from *v* is likely associated with viscoelastic effects in the substrate^46^, while the increase from *d*/*R* is likely due to the increase in contact area. By looking at a single *v* to compare the two cases (e.g. yellow open vs. yellow closed points at 10 µm s^−1^), we find that ${F}_{{{{{{\rm{peak}}}}}}}$ is higher for creasing cases compared to the non-creasing cases. On the other hand, the observation of creasing versus non-creasing between the two types of samples (i.e., aged in ambient light vs freshly prepared) is consistent across two decades of velocities. This implies that the formation of creases is itself not rate-dependent, although the rate can affect the measured forces. These unique experimental observations motivate the following questions: (1) What governs the formation of creases? Because the experiments presented in Fig. 2 exhibit higher ${F}_{{{{{{\rm{peak}}}}}}}$ for creasing samples, it is intuitive to assume that there is an increase in substrate-particle adhesion with light aging, which leads to the formation of creases. (2) How does the crease progress through the contact zone and release? Unlike the air pocket in Schallamach waves, the creases observed in our experiments exhibit self-contact and it is intriguing to ask whether the ruck through a rug mechanism is still applicable to a self-contacting crease.

### Interfacial strength governs crease formation

**Fig. 3:**
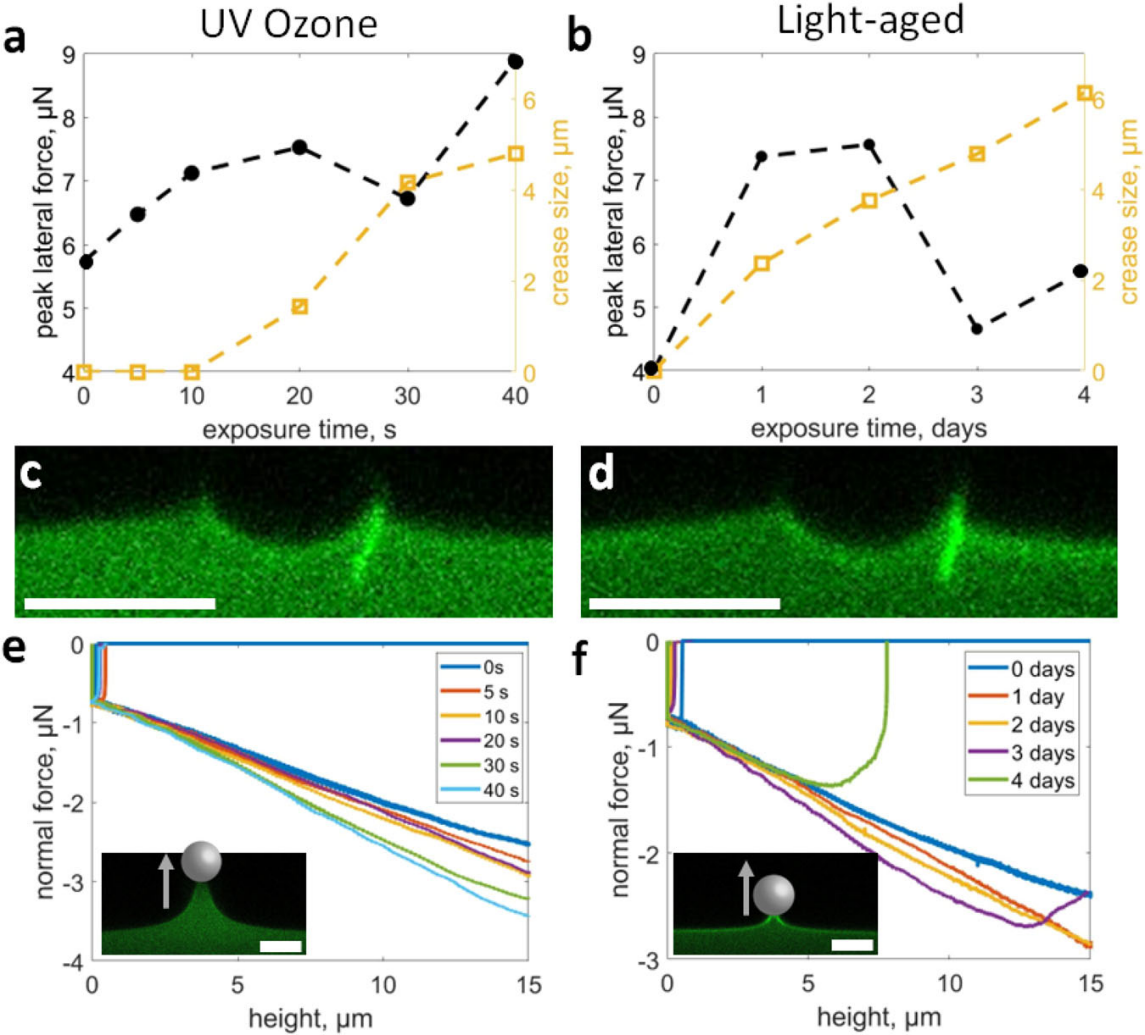
Peak lateral force, crease size, and normal adhesion with surface treatments. **a** Crease size and ${F}_{{{{{{\rm{peak}}}}}}}$ as a function of increasing UV ozone time. **b** Crease size and ${F}_{{{{{{\rm{peak}}}}}}}$ as a function of increasing time in ambient light conditions. **c** A crease on a 40 s UVO-treated surface. **d** A crease on a 3-day light-aged surface. **e** Vertical adhesion force vs. height curves of a surface exposed to UVO for different times. Inset: Confocal image showing maximum vertical extension for a 40 s UVO treated surface. **f** Vertical adhesion force vs. height curves of a sample with different light-aging times. Inset: Confocal image of maximum vertical extension for a 4-day light-aged sample. Scale bars: 20 ${{{{{\rm{\mu }}}}}}{{{{{\rm{m}}}}}}$.

With our hypothesis that higher substrate-particle interaction leads to the initial formation of creases, we set out to test this prediction experimentally. One way to increase the adhesion of PDMS to glass is by short-duration UV–ozone treatment (UVO), which we anticipated to be easier to control than ambient light-aging^47^. Therefore, freshly prepared PDMS samples are UVO-treated from 0 to 40 s; this range is expected to have a surface modification depth of the order ~1 nm^44^, as opposed to longer UVO treatments (~10 min or more) that create a thicker glassy layer^48^. Upon lateral testing at $d/R=0.1$ and $v=1\,{{{{{\rm{\mu }}}}}}{{{{{\rm{m}}}}}}$ s^−1^, samples treated for 10 s or less show no creases (Fig. 3a). For samples treated for 20 s or more, creases appear and increase in size with increasing exposure. Crease size is defined here as the length of the self-contacting crease before it is pulled underneath the microsphere, prior to any crease distortion. To compare UVO-treated surfaces with light-aged surfaces, we conducted the same set of experiments but with aging in ambient light conditions over the course of 1–4 days. Creases appear on light-aged samples after 1 day and continue to grow in size for up to 4 days (Fig. 3b) (recall the data in Fig. 2 is light-aged for 4 days). The creasing behavior in the UVO and light-aged surfaces are similar, with creases that grow with increasing exposure. For comparison, a crease after 40 s of UVO treatment (Fig. 3c) appears nearly identical to a crease after 3 days of light-aging (Fig. 3d). In addition, increasing exposure time in both cases initially increases *F*_peak_; however, a dip occurs once the crease size reaches ~4 μm (i.e. 30 s UVO and 3-day light aging). This non-monotonic behavior suggests an intricate coupling between crease initiation and lateral force: Although nucleation of creases requires a high lateral force, further growth of the creases can lower ${F}_{{{{{{\rm{peak}}}}}}}$.

Since creases occur on both UVO and light-aged samples, we sought to confirm an increase in adhesion for both cases. Normal indentation and pull-off tests are employed with the same microsphere (Fig. 3e). The pull-off forces measured from these tests can serve as a general metric for comparing the adhesion between the microsphere and the UVO or light-aged substrates. Unfortunately, the freshly prepared samples and UVO-treated samples display a pull-off distance beyond the 15 ${{{{{\rm{\mu}}}}}} {{{{{\rm{m}}}}}}$ vertical limits of our AFM. However, the force–height data are at least consistent with the expectation of increasing normal adhesion. Surprisingly however, the pull-off force and the work of separation (area under the curve) decrease with increased aging for the light-aged samples (Fig. 3f). The different roles of adhesion for the UVO and light-aged samples, despite the similar crease morphology, signals that the picture of adhesion being a generic factor controlling the nucleation of creases is too simplistic.

**Fig. 4:**
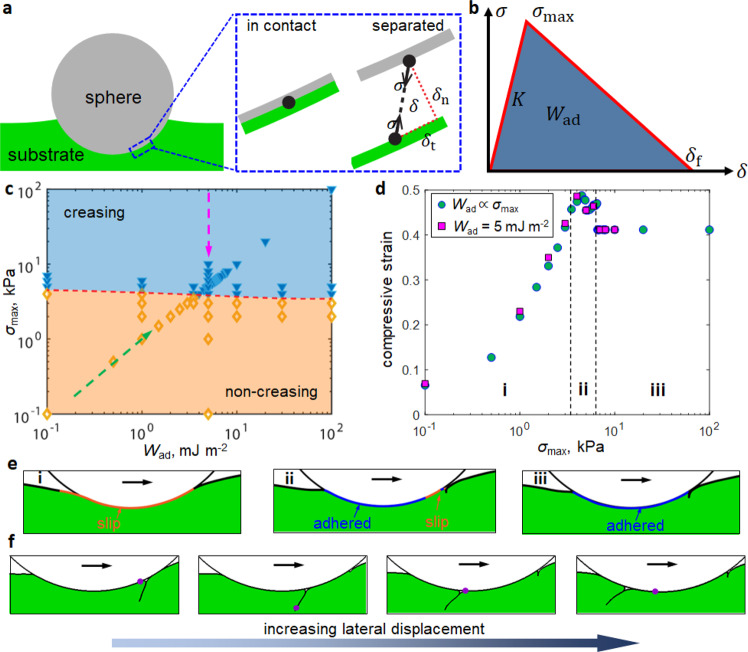
FEA simulations of crease nucleation and progression. **a** Schematic illustration of the contact between the rigid microsphere and the substrate (left). The inset on the right shows a zoomed-in view of the interface in its initial state when contact is just established, and the separated state where the two surfaces are relatively displaced and thus a traction $\sigma$ and separation $\delta$ are used to define the interfacial adhesion. ${\delta }_{{{{{{\rm{n}}}}}}}$ and ${\delta }_{{{{{{\rm{t}}}}}}}$ are the normal and tangential separations, respectively. **b** A cohesive zone model with bilinear traction-separation law is used to define the adhesion energy ${W}_{{{{{{\rm{ad}}}}}}}$ on the interface. **c** Phase diagram for creasing in terms of the adhesion energy ${W}_{{{{{{\rm{ad}}}}}}}$ and interfacial strength *σ*_max_. All simulations were conducted by modeling the substrate as a soft elastic solid (Supplementary Note 2). **d** Maximum compressive strain in the substrate versus the prescribed ${\sigma }_{{{\max }}}$ under either fixed ${W}_{{{{{{\rm{ad}}}}}}}$ (square symbols) or varying ${W}_{{{{{{\rm{ad}}}}}}}$ (circular symbols). **e** Interface conditions corresponding to the three regimes shown in (**d**). **f** Simulation snapshots showing the progression of a crease through the contact region using ${W}_{{{{{{\rm{ad}}}}}}}$ = 8 mJ m^−2^ and ${\sigma }_{\max }=8\,{{{{{\rm{kPa}}}}}}$ and assuming no self-adhesion within the crease. The purple dot represents a material point on the substrate surface.

Creasing is by nature an instability of substrate deformation triggered by a critical compressive strain^49^. Although in our experiments the substrate was not subjected to any global compression, the microsphere generates local compression in the substrate ahead of its leading front. Since the nucleation of creases does not involve a characteristic length scale^34^, we hypothesize that creases nucleate in the local compressive region when the maximum compressive strain reaches a critical value. To demonstrate, we use finite element analysis (FEA) (Supplementary Notes 2 and 3) to qualitatively simulate the contact between the microsphere and substrate. Motivated by the observation that the formation of creases is rate-independent (Fig. 2d), here we model the substrate as an elastic neo-Hookean solid and the microsphere as a rigid body. Finite strain within the substrate is considered which is necessary for simulating the highly localized large deformation around a crease. The microsphere-substrate adhesion is represented by a cohesive zone model featuring a bilinear function relating the magnitude of the attractive traction *σ* resisting separation to the relative separation *δ* between two points initially in contact (Fig. 4a). Such bilinear cohesive zone model has been extensively applied to simulate adhesion^50,51^ and recently to study Schallamach waves^52^. It can be viewed as an approximation of the attractive portion of the van der Waals interaction between two surfaces. The area underneath the triangle defined by $\sigma (\delta )$ (Fig. 4b) represents the mechanical work required to separate a unit area of contacting interface, also known as the adhesion energy *W*_ad_. This parameter is directly related to the adhesion force in various mechanical tests (e.g., indentation or peel tests), and hence is often used as the metric to quantify adhesion. Apart from *W*_ad_, the cohesive zone model also includes additional parameters, i.e., the maximum traction *σ*_max_ and the final separation *δ*_f_. In particular, *σ*_max_ represents the highest stress that can be sustained by the adhesive interface and will be referred to as the interfacial strength hereafter.

We use the FEA model to examine which cohesive zone parameter controls the nucleation of creases. Given that *W*_ad_ = *σ*_max_ *δ*_f_/2, we can independently vary *W*_ad_ and *σ*_max_ as long as *δ*_f_ can shift accordingly. Specifically, we either hold *δ*_f_ constant so that *W*_ad_ is proportional to *σ*_max_, or increase *σ*_max_ while holding *W*_ad_ constant, where *δ*_f_ decreases accordingly. The rest of the cohesive zone parameters, specifically the initial slope *K*, are held constant. The result in Fig. 4c shows that the onset of creasing is governed by *σ*_max_ and insensitive to *W*_ad_. This is not surprising since the maximum compressive strain in the substrate should scale with the tangential traction within the contact region, which is governed by *σ*_max_ rather than *W*_ad_. In Fig. 4d, we plot the maximum compressive strain in the substrate versus the prescribed *σ*_max_ under either fixed or varying *W*_ad_. Note that the maximum compressive strain refers to the normal strain component along the horizontal direction and is extracted as the highest compressive value observed spatially and temporally in a simulation (Supplementary Note 4). As expected, the relation between maximum compressive strain and *σ*_max_ is not affected by *W*_ad_. More interestingly, we observe three regimes as *σ*_max_ is increased (Fig. 4d, e). When *σ*_max_ is small (i.e., Regime i), the compressive strain is insufficient to trigger crease nucleation and the entire interface undergoes slip. When *σ*_max_ is large (i.e., Regime iii), the interface remains adhered and a crease is nucleated at the front of the microsphere. Creasing caps the maximum compressive strain at ~40%, regardless of how large is *σ*_max_. This level of maximum compressive strain is close to the critical creasing strain (~35%) for a thick substrate under global uniaxial compression^49^. Between the two limiting regimes there is an intermediate one (Regime ii) where a crease nucleates, but partial slip occurs in a small region next to the crease. The FEA results support our hypothesis that crease nucleation is modulated by the local compressive strain in the substrate, which is further governed by the interfacial strength. This finding helps resolve the seeming paradox observed in Fig. 3, since the pull-off force during a normal indentation test seems to be connected with *W*_ad_ rather than *σ*_max_^53^.

FEA simulations also reveal the mechanism of how a crease moves across the contact region (Fig. 4f). After nucleation, the crease first grows as the lateral displacement of the microsphere continuously increases. Unlike symmetric creases formed under global compression, in our simulations the crease tilts toward the trailing edge of the microsphere due to the asymmetric local compression; this is consistent with experimental observation (Fig. 2a3). Such asymmetry results in a Schallamach-wave-like motion of the crease: Its left edge is peeled from the microsphere while its right edge makes contact with the microsphere, as demonstrated by the relative position of the crease and a material point highlighted in Fig. 4f. This mechanism enables the crease to progress through the contact zone while maintaining the adhesive interface between microsphere and substrate, thereby avoiding a catastrophic drop of lateral force. Indeed, our experiments show that the lateral force continues to rise during crease motion and drops only after the crease moves out of the contact region (Fig. 2c).

### How self-adhesion inside the crease relates to progression and release

Self-adhesion between the two contacting surfaces within a crease is evident in our experimental system. As shown in Fig. 2a4, a segment of the crease is retained even after it moves out of the trailing edge. Given that the substrate is under tension at the trailing edge of the microsphere, there must be self-adhesion within the crease to resist opening under tension. This motivates a unique question: How does self-adhesion affect the Schallamach wave-like motion of a crease? A conventional Schallamach wave features an air pocket for which self-adhesion is irrelevant. In the literature on creasing, it has been recently discovered that self-adhesion can resist the full recovery of a symmetric crease when the global compression is removed, leaving a scar on the substrate^35^. However, the Schallamach wave-like motion of an asymmetric crease involves more complex deformation in the substrate than the unfolding of a symmetric crease. Since self-adhesion is not included in the FEA model used for Fig. 4, we use FEA simulations to obtain physical insights into the role of self-adhesion (Supplementary Note 5). Here we apply a visco-hyperelastic model calibrated by rheological data to the substrate (Supplementary Note 2) since viscoelasticity may affect the crease motion, as indicated by the rate-dependent peak lateral forces in Fig. 2d.

**Fig. 5:**
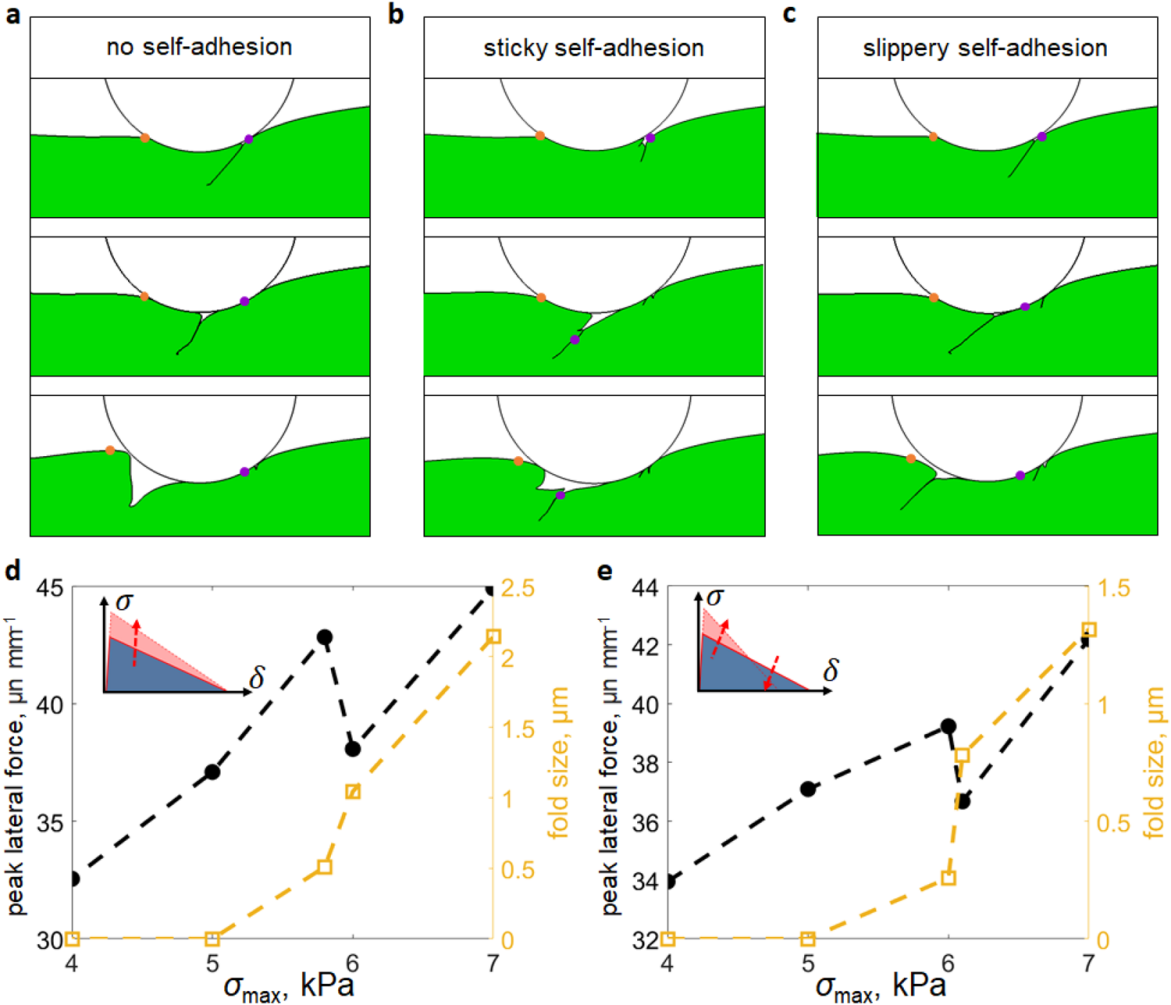
Effects of self-adhesion on the Schallamach-wave-like motion of a crease. **a**–**c** Simulation snapshots showing the motion of a crease under **a** no self-adhesion, **b** sticky self-adhesion and **c** slippery self-adhesion, as defined in Supplementary Note 5. The orange and purple dots represent two material points originally located at the leading front and trailing edge, respectively. The crease in **c** is Schallamach wave-like and does not open until passing through the contact zone, resembling experiments. **d** and **e** Peak lateral force and crease size versus interfacial strength ${\sigma }_{{{\max }}}$ under **d** a fixed ${\delta }_{{{{{{\rm{f}}}}}}}$ = 2 μm and hence varying ${W}_{{{{{{\rm{ad}}}}}}}$, and **e** a fixed ${W}_{{{{{{\rm{ad}}}}}}}\,$= $5\,{{{{{\rm{mJ}}}}}}\,{{{{{{\rm{m}}}}}}}^{-2}$. All simulation data in **d** and **e** are obtained with slippery self-adhesion in the crease, and the substrate is assumed to be a soft viscoelastic solid (see Supplementary Note 2) with a sliding velocity of $v$ = 0.1 μm s^−1^.

Simulation results for three cases with different self-adhesion conditions are shown in Fig. 5a–c. Although the case with no self-adhesion (Fig. 5a) can capture Schallmach wave-like crease motion, discrepancy arises as the crease approaches the trailing edge of the microsphere. Instead of remaining in contact as observed in experiments, the crease opens up due to the local tensile region near the trailing edge. This is consistent with the expectation that a crease cannot remain folded under tension if adhesion is absent. In the second case (Fig. 5b), we implement identical self-adhesion interactions along both normal and tangential directions within the crease interface. As a result, the self-adhesion not only prevents the crease from opening but also resists relative slippage along the crease, and hence is referred to as the sticky self-adhesion. Interestingly, the sticky self-adhesion completely suppresses the Schallamach wave-like motion. Instead, for the crease to progress through the contact zone, the entire adhesive interface behind the initial crease must debond, as demonstrated by the relative positions of two highlighted material points in Fig. 5b. Physically, for the Schallamach wave-like crease motion to be possible, material points on the two sides of a crease must move in the opposite direction (see Fig. 5a), which inevitably leads to slippage along the crease. This finding motivates us to pursue the third case (Fig. 5c), referred to as the slippery self-adhesion. In this case, self-adhesion is set primarily along the normal direction of the crease and much reduced along the tangential direction, such that the crease surfaces are under adhesive yet slippery contact. In this case, the crease undergoes a Schallamach wave-like motion while growing in size and remains closed when it reaches the trailing edge, which is most consistent with our experiments (Fig. 2 and Supplementary Note 6). In addition to the crease formation, the experimental force trends in Fig. 2 are qualitatively captured by the simulations (Supplementary Note 6).

## Discussion

The combination of self-adhesion and free slippage within the crease is unusual but not unfounded. For example, liquids in a swollen surface can lead to adhesion in the normal direction and yet allow for slippage^54^. For PDMS materials, slip can be defined locally on the molecular scale, which has been demonstrated using both polymer melts and elastomers;^55^ this is consistent with the idea that self-slip occurs in our very soft elastomer within the microscale crease. In addition, Newby et al. demonstrated that normal adhesion and slippage can be separately controlled by the surface energy and mobility of self-assembled monolayers, respectively^56^. Moreover, similar concepts of slip near soft peeling fronts have been observed^57^. We acknowledge that it is difficult to quantify the self-adhesion and self-slip within our creases, which should require careful characterization in future efforts. Consequently, quantitative agreement between simulations and experiments is not pursued due to challenges in obtaining calibrated adhesion parameters. Yet, our simulation results of the FEA with slippery self-adhesion are qualitatively consistent with the main experimental observations. As a demonstration, we plot the peak lateral force and crease size versus the interfacial strength *σ*_max_ between the microsphere and substrate under either increasing adhesion energy *W*_ad_ (Fig. 5d) or fixed *W*_ad_ (Fig. 5e). Assuming both UVO treatment and light-aging in Fig. 3 increase ${\sigma }_{{{\max }}}$ but affect ${W}_{{{{{{\rm{ad}}}}}}}$ differently, the trends of simulation results in Fig. 5d, e are similar to as those in Fig. 3a, b. In particular, the non-monotonic dependence of peak lateral force on crease size in Fig. 3a, b is captured by the simulations.

The creases in our study are reminiscent of both surface creases found during symmetric global compression as well as Schallamach waves observed in macroscale, rubber tribology. Therefore, it is instructive to consider the similarities and distinctions of our creases to these phenomena. Self-contacting surface creases occur when a global compression is applied to a surface; for example, creases arise when attaching a soft layer to a pre-strained surface and releasing^36^, or simply applying a compressive strain on a soft block^58,59^. Crease nucleation has been calculated to be similar to a first-order transition^59^, and also sensitive to local imperfections on the surface^60^. Although confocal microscopy images of creases in the literature resemble our creases^35^, they have a different curvature at the surface. Moreover, near the elastocapillary length, defined as a ratio of surface tension and elastic modulus, *L*_EC_ = *γ*/*E*, creases can leave scars after removal of strain due to capillarity and self-adhesion. In our case, *L*_EC_ ≈ 4 μ*m*, assuming *γ* = 20 mN m^−1^ and *E* = 5 kPa. Yet no scars are observed once the self-contacting crease moves through the contact zone. The lack of scaring may be due to low adhesion hysteresis but, more likely, is due to the state of tension at the trailing edge that pulls the crease open. However, it is interesting to note that the dip in *F*_peak_ (Fig. 3) occurs when the crease size is near ${L}_{{{{{{\rm{EC}}}}}}}$, which may suggest a size-dependent force dip. Our creases are similar to Schallamach waves, where a surface instability manifests from a build-up of compressive stress at the front, which then passes through the contact zone^61^. In contrast to conventional Schallamach waves, however, our creases are self-contacting such that no air gap is visible. The crease maintains a self-contacting profile until it reaches the trailing edge and opens from the local tension. Additionally, the crease geometry is relatively stable until it is near the trailing edge; we can stop the microsphere for several minutes without the crease moving (Fig. 2c–e). Hence, the creases discovered here bridge the physical mechanisms of creases formed from global compression and Schallamach waves.

From an experimental standpoint, the small size of our microsphere facilities using confocal microscopy to observe a single crease moving through the contact. For larger size scales, it would be challenging to see both sides of the sphere. Moreover, it would be difficult to control the contact size. For example, if a large glass sphere is brought into contact, the soft surface would deform to increase the contact area to much larger than ${L}_{{{{{{\rm{EC}}}}}}}$. Additionally, our investigation reveals the significance of silicone processing/aging on the interfacial mechanics of soft contacts. Changes in mechanical and surface properties have been observed over aging time^43^, with different curing processes^62^, and with UV or UVO exposure^44^. In addition, soft materials, like lightly crosslinked PDMS, often have uncrosslinked free chains that are not tethered to the polymer network. We previously found that the PDMS used here (60:1 Sylgard 184) contains nearly 60% extractable materials^41^. These mobile molecules have been shown to form a liquid meniscus at the contact line of a stationary drop or microparticle^38,40,63^. In our case of a moving microparticle, the free chains are expected to reduce ${\sigma }_{{{\max }}}$ by acting as a partial lubricant, lowering but not eliminating the network–particle contact^37,55,64–67^. At the same time, free chains can aid in maintaining high normal adhesion due to capillarity;^68^ we associate this case with freshly prepared, untreated surfaces. However, minor stick–slip behavior may result from a mixed contact interface, where the particle is partially in contact with the network and partially in contact with free chains (free mobile molecules) or dangling ends, which may also be the case for hydrogels^7,37,65,69–71^. After ambient light-aging (Fig. 2a), a line of increased fluorescence is observed at the surface. We also notice that the fluorescent signal of our fluorescent monomer increases after crosslinking into the network (Supplementary Note 7, Supplementary Fig. 7). Therefore, the increased fluorescence may suggest some additional crosslinking at the surface. This idea is not completely new; for example, UV light has been suggested to create smoother surfaces with increased crosslinking at the surface^44,45^. Higher radiation has also been reported to lead to crosslinking and scission but with predominant crosslinking^72^. This crosslinking would reduce the amount of uncrosslinked free chains and dangling free ends near the surface that could partially lubricate the contact. Although this crosslinking may lead to a marginal increase in modulus at the surface, a stiff layer is not required for creasing^73^. We confirmed this by running a simulation with a stiff layer, which illustrates that a higher interfacial strength is required to crease with an increasingly stiff layer (Supplementary Note 7, Supplementary Fig. 8).

In summary, we introduce a unique approach to experimentally investigate how a microscopic contact starts to move laterally on a soft, adhesive surface. Using lateral force and confocal microscopy, we show that both creasing and non-creasing cases can occur. Creasing emerges with sufficiently high interfacial strength between the particle and the surface, which manifests experimentally through light-aging or UVO exposure. By tuning the parameters that govern adhesion energy in simulations, we conclude that the interfacial strength plays a dominating role in crease formation. Most interestingly, a crease moves through the contact zone in a Schallamach wave-like manner, fully opening only on the trailing edge due to local tension. This requires that the self-contacting interface within a crease is adhesive yet slippery. Future efforts should consider experiments that are able to tune self-adhesion vs. self–slip interfaces independently, potentially through molecular architectures, compositions, or multi-phase materials.

## Methods

### Materials

Dow Sylgard 184 was purchased from Ellsworth Adhesives as a two-part kit. Polydisperse soda lime glass microspheres (2.5 g cc^−1^) were purchased from Cospheric LLC. No. 1 glass coverslips and chloroform were purchased from VWR, and fluorescein diacrylate was purchased from Sigma-Aldrich.

### PDMS (polydimethylsiloxane) preparation

Sylgard 184, a commercially available polydimethylsiloxane two-part kit, was dyed with fluorescein diacrylate. The two parts were mixed with a 60 to 1 ratio, giving a modulus of 3.5 ± 0.5 kPa. The material was prepared following a previously described procedure. Briefly, fluorescein diacrylate was first dissolved in chloroform, mixed with Sylgard 184 base, and left for the chloroform to evaporate at 65 °C. The curing agent was subsequently mixed with the dyed base^37^. The mixture was degassed under a vacuum to remove trapped air for ~30 min and spin-coated on a glass coverslip at 1000 RPM for 60 s to achieve a thickness of ~75 µm. Other RPMs were used to increase or decrease the thickness during control experiments to test the effect of sample thickness^37^. An RPM of 1000 was chosen to maximize the thickness of the PDMS while maintaining the necessary resolution using an optically correctable objective on the confocal microscope. After spin-coating, the coverslip with the uncured PDMS was placed in an oven at 65 °C for 48 h to cure.

### Characterization

#### Imaging via confocal microscopy

Images were taken of the fluorescein diacrylate dyed samples using an inverted Leica confocal microscope equipped with a piezo objective holder that allows for fast cross-sectional scans and a ×40 air objective. A correction ring on the objective enabled focusing on the air-PDMS interface through the thickness of the PDMS sample. To take *x*–*z* images, the piezo was used to capture a single slice through the *x*–*z* plane. For the full 3D image in Fig. 1e, the probe was stopped, such that a full 3D scan can be captured. However, most images were taken as a single *x*–*z* slice to increase frame rates and used for analysis. Variable imaging rates and resolutions were used to optimize the imaging rate and quality. An excitation laser with a wavelength of 488 nm and a collection range of 495–520 nm was consistently used. Note that the laser power was left constant when comparing the fluorescence signal before and after crosslinking.

#### Image analysis

The confocal images were analyzed using ImageJ. A sphere was fit to the shape of the colloidal probe in the image to determine the location. To record the indentation depth, the distance from the lowest point of the sphere to the PDMS surface far outside the contact zone was measured, while the particle was at rest.

#### Force microscopy

A JPK Nanowizard 4 was mounted on top of the confocal microscope. A colloidal probe AFM cantilever was used for all measurements. *A* ~ 17 µm diameter glass sphere was attached to a thermal noise-calibrated ACL-TL tipless cantilever having a nominal spring constant of ~40 N m^−1^ using high-strength epoxy. For lateral force tests, the microsphere was indented into the PDMS at a rate of 1 µm s^−1^ and held for up to 45 s. To conduct lateral force measurements, a motorized, high-precision linear stage (Physik Instrumente, L-509), mounted to the side of the confocal microscope, was connected to a custom sample holder between the AFM head and the confocal microscope. This setup enables lateral translation of the sample via the motorized linear stage, while the confocal microscope objective and the colloidal probe of the AFM remain aligned and in focus. Prior to these experiments, lateral force calibration of the cantilever was performed by scanning the tip across a clean glass slide at different normal loads. Based on a procedure in the literature, we assumed a coefficient of friction of 0.4 and calculated the frictional force relative to the cantilever deflection^74^. For normal adhesion tests, the microsphere was indented into the surface at a rate of 0.1 µm s^−1^ to a depth of ~0.2 µm, followed by retraction at the same rate. To test if creasing behavior is affected by the dye itself, we conducted experiments without dye but in reflection mode on the confocal microscope. Although this imaging is not suitable to measure details, fold release can still be observed (Supplementary Movies 3 and 4).

### Simulations

Finite element analysis (FEA) was performed to simulate the microscale indentation and sliding experiments using the commercial package ABAQUS (version 2020, Simulia, Providence, RI, USA). The FEA model consisted of two components: a rigid sphere and a soft substrate. To reduce the computational cost, a two-dimensional (2D) plane strain model was built where the spherical microsphere was modeled as a rigid circle with a diameter of 17 µm, and the substrate was a 200 µm × 50 µm rectangle meshed with 2D plane strain elements (CPE4RH). The substrate was subjected to a fixed boundary condition at its bottom surface and traction-free boundary conditions at its left, right and top surfaces. In addition, the top surface of the substrate can form adhesive contact with the microsphere. The contact between the microsphere and the substrate was modeled as hard contact and was implemented through the direct method that strictly enforces the pressure-overclosure relationship of hard contact. This contact formulation generates a compressive contact pressure on the interface between the microsphere and the substrate, which should be distinguished from the attractive adhesion prescribed by the cohesive zone model described next. Cohesive zone modeling with the bilinear traction-separation law was adopted to describe adhesion between the microsphere and the substrate as well as self-adhesion of the substrate surface. Reversible cohesive zones were implemented to allow re-establishment of adhesion after complete separation. More information on the material model, cohesive zone, and mesh convergence test is provided in Supplementary Note 2. Initially, the microsphere was on top of the substrate surface with a 1 µm gap between the microsphere and the substrate surface. In a simulation, the microsphere was first moved downwards at a velocity of 1 µm s^−1^ until a desired indentation depth was achieved. After a 17-second relaxation step (to account for the experimental time gap between normal indentation and lateral motion), the microsphere was moved horizontally with the indentation depth held fixed. All simulations were performed using a dynamic/implicit solver to accommodate the instability associated with creasing. Since physically the 2D plane strain model represents the cross-section of an infinitely long cylinder in contact with the substrate, the lateral force obtained from the simulations is a line force, i.e., force per unit length along the out-of-plane direction.

## Supplementary information

Supplementary Information

Peer Review File

Description of Additional Supplementary Files

Supplementary Movie 1

Supplementary Movie 2

Supplementary Movie 3

Supplementary Movie 4

## Data Availability

The data that support the findings of this study are available from the corresponding author upon request.
