# Supplementary material for: Creasing in microscale, soft static friction
Source: Nat Commun. 2023 Apr 24;14:2362. doi: 10.1038/s41467-023-38091-7 (PMC10126204; doi:10.1038/s41467-023-38091-7)
Supplement: Supplementary file 1 — Supplementary Information [file 41467_2023_38091_MOESM1_ESM.pdf]

Supplementary Information for: **Creasing in microscale, soft static friction**

Justin D. Glover[1†], Xingwei Yang[2†], Rong Long[2], Jonathan T. Pham[1,3*]

[1] Department of Chemical and Materials Engineering, University of Kentucky, Lexington, KY 40506, USA

[2] Department of Mechanical Engineering, University of Colorado Boulder, Boulder, CO 80309, USA

[3] Department of Chemical and Environmental Engineering, University of Cincinnati, Cincinnati, OH, 45221, USA

†Equal contribution.

*Email: Jonathan.Pham@uc.edu.

Supplementary Movies:

Supplementary Movie 1: A movie showing the creasing case. The creasing force data from this movie is plotted in Figure 2c of the main text. Lateral motion started at ~18 s. The substrate is being pulled to the left.

Supplementary Movie 2: A movie showing the non-creasing case. The non-creasing data from this movie is plotted in Figure 2c of the main text. Lateral motion started at ~45 s. The substrate is being pulled to the left.

Supplementary Movie 3: A movie showing the non-creasing case in reflection mode. Lateral motion started at ~15 s. The substrate is being pulled to the left.

Supplementary Movie 4: A movie showing the creasing case in reflection mode. Lateral motion started at ~33 s. The substrate is being pulled to the left. Though the crease is difficult to visualize in reflection, a small dip down of the surface can be seen at the front of the probe, which is more similar in geometry to the creasing case than the non-creasing case. Additionally, the release at the back of the probe appears larger. These observations suggest that crease formation is not due to the presence of dye in the sample. Moreover, it suggests that creasing is not caused by the dye crosslinking to the network due to light exposure.

Supplementary Note 1: Control experiments for creasing

As discussed in the main text, we found that samples left to age in ambient light at room temperature, after the curing process, exhibited creasing behavior. This supplementary note describes a range of different experimental controls used to conclude that creases appear after light-aging. These variables include the Sylgard 184 mixing ratio (modulus), particle size, varying dwell times, sample thickness, and extended curing times; however, creasing was consistently on a per sample basis. In other words, light-aged samples always exhibited creases while freshly prepared samples did not crease. To test if slight modulus variations, which can occur in batch-to-batch sample preparation, caused creasing, we prepared samples with both 50 and 70 to 1 mixing ratios of Sylgard 184; these possess slightly higher and lower modulus respectively. However, no creases were observed on the freshly prepared samples. Hence, we kept our samples constant at the 60:1 mixing ratio. Two probe sizes were also tested including R = 8.5 and 13.5 μm; again, no creases were observed on freshly prepared samples. To ensure that the creasing is not due to slight variations in dwell time, we varied the dwell time from 0 to 30 seconds; creasing was still not observed on fresh samples. The sample thickness of the PDMS substrate was also confirmed to not be the cause of creasing, since both creasing and non-creasing behavior was observed on samples of the same thickness from ~30 μm to ~90 μm. To consider if extended cure times lead to creasing, we cured samples from 24 hours to one week at 65 °C. After one week in a dark oven, no creases were observed. After conducting these control experiments, we find that freshly prepared samples never crease due to slight modulus variations, sample thickness, particle size, curing time, and dwell time (within the ranges tested). Within our experimental error range, we also do not find that the dye affects the creasing behavior; creases appear to form with the same conditions with and without dye (Supplementary Movie 4). Additionally, the force curves are generally consistent, as illustrated in Supplementary Figure 1.

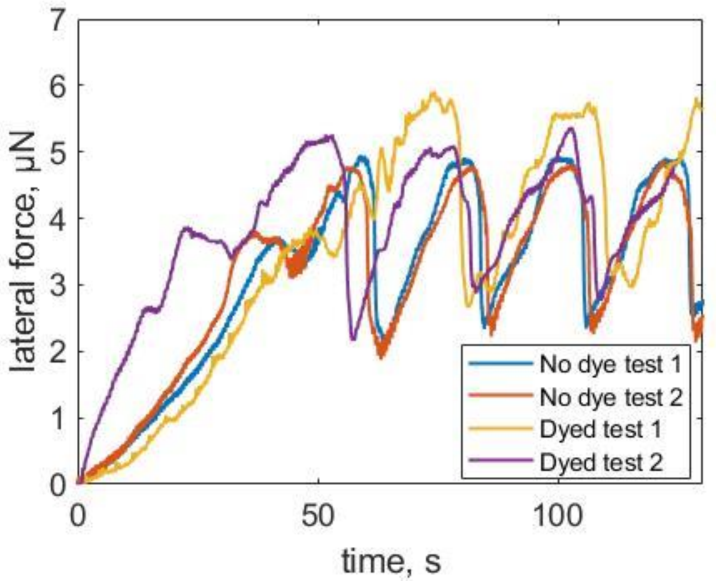

Supplementary Figure 1: Lateral force vs. time curves for two randomly chosen samples with fluorescent dye and two randomly chosen samples with no dye. The peak forces (e.g. presented in Fig. 2 of the main text) are within experimental error.

Supplementary Note 2: Finite Element Analysis (FEA)

All FEA simulation results presented in the main text were generated using a two-dimensional (2D) plane strain model consisting of a rigid indenter and a deformable substrate. The simulations were conducted using a software package ABAQUS (version 2020, Simulia, Providence, RI, USA). This supplementary note elaborates three aspects of the FEA model: i) the material model for the deformable substrate, ii) the cohesive zone model for the adhesion between indenter and substrate, and iii) the mesh convergence tests.

Material model

The PDMS substrate was modelled as an incompressible visco-hyperelastic model that combines the neo-Hookean solid and a Prony series to capture viscoelasticity under finite deformation. This model, based on the framework of Simo,[1] extends the formulation of linear viscoelasticity to accommodate finite deformation kinematics and hyperelasticity, and is readily available in ABAQUS. Since the incompressible neo-Hookean solid is characterized by only the shear modulus, the corresponding visco-hyperelastic model can be fully specified by the relaxation function, which is given below according to the Prony series:

$$G(t) = G_\infty + \sum_{i=1}^{N} G_i \exp(-t/\tau_i), \tag{S1}$$

where G_∞ is the long-term shear modulus in the fully relaxed limit, N is the number of relaxation modes, G_i and τ_i are the shear modulus and relaxation time associated with each relaxation mode, respectively. In the frequency domain, Eq. (S1) results in the following functions of the storage shear modulus G' and the loss shear modulus G'':[2]

$$G'(\omega) = G_\infty + \sum_{i=1}^{N} G_i \frac{\omega^2\tau_i^2}{1+\omega^2\tau_i^2}, \tag{S2}$$

$$G''(\omega) = \sum_{i=1}^{N} G_i \frac{\omega\tau_i}{1+\omega^2\tau_i^2}, \tag{S3}$$

where ω is the angular frequency (unit: rad/s). The parameters G_∞, G_i and τ_i (i = 1, 2, ... N) were calibrated by fitting rheology data of the PDMS substrate using Eq. (S2) and Eq. (S3). Supplementary Figure 2 shows the fitted curves together with the experimental data. The long-term shear modulus G_∞ is found to be 1.89 kPa and the other fitting parameters are listed in Table 1. Under extremely low lateral velocity ($v \to 0$), the viscoelastic substrate is expected to be in its long-term relaxed limit and hence behaves as an elastic solid. The experimental observation that the formation of creases is insensitive to lateral velocity (main text Fig.2d) suggests creasing does not rely on viscoelasticity. Therefore, when studying crease nucleation (main text Fig. 4) we assumed the substrate to be elastic by simply removing the relaxation function, which recovered an incompressible neo-Hookean solid with a shear modulus of G_∞ = 1.89 kPa for the substrate. The full visco-hyperelastic model was applied to the substrate when studying the progression of creases and the corresponding peak lateral force (main text Fig. 5 and Supplementary Figure 6), under the expectation that viscoelasticity may play a role here as indicated by the rate-dependent peak lateral forces (main text Fig. 2d).

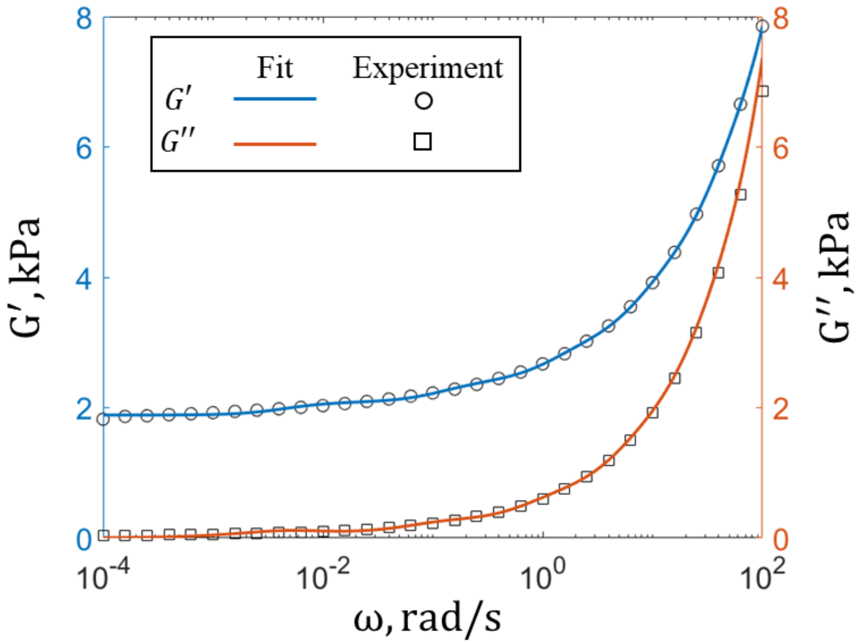

Supplementary Figure 2: Storage (G') and loss (G'') shear moduli as a function of the angular frequency ω. Symbols represent experimental data (circle: G'; square: G'') from rheological tests of the PDMS substrate. Solid lines represent fits (blue: G'; orange: G'') of the Prony series. The rheological data were obtained by shear rheology using a ~1 mm thick, 60 to 1 Sylgard 184 sample between 25 mm parallel plates in the linear regime.[3]

Supplementary Table 1: Calibrated parameters for the relaxation in Eq. (S1) with G_∞ = 1.89 kPa.

i	1	2	3	4	5	6
G_i (kPa)	31.3	2.65	0.324	1.207	0.2	0.617
τ_i (s)	0.0021	0.249	8.0712	0.1264	221.923	0.7953

Cohesive zone

Adhesion between the indenter and the substrate was captured using a cohesive zone model. This model prescribes a traction-separation relation between the two interfaces,[4] as schematically illustrated in Fig. 4a. Briefly, when two contacting points on the indenter and the substrate are separated by a vector of $\boldsymbol{\delta}$, they are subjected to an attractive traction $\boldsymbol{\sigma}$ (force per unit area) induced by adhesion. Both vectors of $\boldsymbol{\sigma}$ and $\boldsymbol{\delta}$ can be resolved to components along the normal and tangential directions of the interface. Note that although 2D interface only has one tangential direction, the cohesive zone model was set up for a general 3D interface with two tangential directions but one of the tangential directions remains inactive for 2D simulations. We assumed an uncoupled and isotropic traction-separation relation, implying that $\boldsymbol{\sigma}$ and $\boldsymbol{\delta}$ are along the same direction. Therefore, the traction-separation law can be specified by relating the magnitudes of $\boldsymbol{\sigma}$ and $\boldsymbol{\delta}$, which are denoted as σ and δ, respectively. This assumption implies that we have neglected any potential mixed-mode effect in the cohesive zone, which has been shown recently to play a role in the contact mechanics under combined normal and shear loadings.[5] However, since we do not have a detailed calibration of the cohesive zone parameters between the indenter and the substrate, we have adopted an uncoupled, isotropic traction-separation relation for simplicity given that our goal to capture the qualitative trend rather than quantitatively reproduce the experimental data.

In general, it is challenging to directly measure the relation between σ and δ. To capture the essential physics, we adopted a simple bilinear traction-separation relation featuring three parameters: the maximum separation δ_f, the interfacial strength σ_max, and the initial stiffness K. Alternatively, the adhesion energy $W_\mathrm{ad} = \sigma_\mathrm{max}\delta_\mathrm{f}/2$ (i.e., the area underneath the traction-

separation curve) is a parameter representing energy required to separate a unit area of the interface. As shown in Fig. 4b, σ first increases linearly with δ with a slope of K. Interface damage is initiated when the interfacial strength σ_{max} is achieved. Specifically, we used the maximum stress criterion for damage initiation, which is stated as:

$$\max\left\{\left\langle\sigma_{\text{n}}\right\rangle, \sigma_{\text{s}}\right\} = \sigma_{\max}, \tag{S4}$$

where σ_{n} and σ_{s} represent the normal and tangential traction components, respectively, and the Macaulay bracket $\langle\bullet\rangle$ is to signify that a compressive normal traction σ_{n} does not initiate damage. Complete interface failure occurs when δ_{f} is reached and the traction reduces to 0. In this work, we used $\delta_{\text{f}} = 2\ \mu\text{m}$ and $K = 2 \times 10^{11}\ \text{N/m}^3$ if not otherwise specified. The values of σ_{max} and W_{ad} were varied to probe the effects of interfacial strength and adhesion energy.

When the crease progresses across the contact region, a portion of the substrate surface may re-engage contact with the indenter after the being completely detached from the indenter. In such cases, adhesion should be reestablished as the substrate surface reenters into contact with the indenter. To allow reestablishment of adhesion after complete separation, we enforce the same cohesive interaction for reformed contact interface after complete separation by selecting the option of "Allow cohesive behavior during repeated post-failure contacts" in the cohesive behavior settings in ABAQUS. This enables the interface damage variable to be reset to zero upon reformation of contact interface, resulting in newly established cohesive zone.

Mesh Convergence

The mesh of the FEA model is shown in Supplementary Figure 3a. The substrate was meshed with 2D plane strain elements (CPE4RH) with uniform mesh size along the horizontal direction and biased mesh size along the vertical direction. The smaller elements were located at the surface of the substrate and were square in shape with an edge length of l_{m}. The indenter was modelled as 1D rigid wire with an element size of 0.1 μm. Recall that the diameter of the indenter was 17 μm. To test if the mesh is sufficiently fine to resolve the highly localized deformation associated

with creasing, we performed a mesh convergence test using a benchmark case where the substrate was elastic (i.e., incompressible neo-Hookean solid with a shear modulus of 1.89 kPa) and was subjected to an indentation depth of $0.4R$ = 3.4 μm (R = 8.5 μm is the indenter radius). The interfacial strength $\sigma_{\max}$ was set to be 8 kPa to enable creasing, which corresponds to an adhesion energy W_{ad} of 8 mJ/m^2 since $\delta_{\mathrm{f}} = 2$ μm. We carried out a series of simulations with the smallest substrate element size l_{m} varying from 4 μm to 0.2 μm, and extracted the peak lateral force and crease size (Supplementary Figure 3b) as a function of the mesh size l_{m}. As mentioned in the Methods section of the main text, the lateral force given by the 2D simulations is a line force (i.e., force per unit length along the out-of-plane direction) and was converted to the total lateral force by multiplying the line force by the indenter diameter. The crease size was defined as half of the contour length from one end of the crease to the other. The data in Supplementary Figure 3c clearly shows that the simulation result, in terms of the peak lateral force and crease size, converges when l_{m} is smaller than 0.4 μm. Therefore, we have adopted l_{m} = 0.2 μm in all of our simulations.

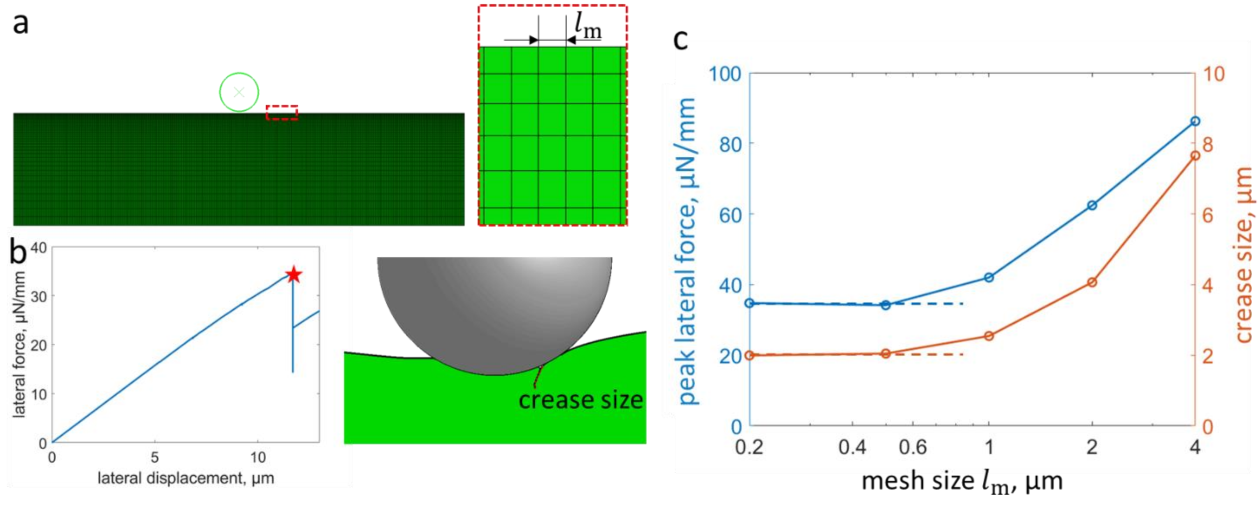

Supplementary Figure 3. Mesh convergence test. (a) Finite element mesh for the 2D model (left). The inset shows a zoomed-in view of the mesh near the substrate surface consisting of square elements with an edge length of l_{m}. (b) Peak lateral force (marked by the red star on the plot of lateral force versus lateral displacement) and crease size obtained from a representative simulation. (c) Peak lateral force per unit length and crease size as a function of the mesh size l_{m} (log-scale). The results converge when l_{m} is less than 0.5 μm.

Supplementary Note 3: 2D versus 3D Model

The 2D plane strain model adopted in our simulations should be interpreted as the cross-section of an infinitely long cylinder in contact with the substrate, which is obviously different from the experimental geometry with a spherical indenter. To shed light on the effect of this difference, we built a 3D model as shown in Supplementary Figure 4a. The substrate was meshed by linear brick elements (C3D8RH). The smallest elements are located at the center of the top surface with an element size of 0.2 μm × 0.2 μm × 0.2 μm, which is similar to the 2D model. The indenter was modeled as a rigid shell surface with an element size of 0.2 μm × 0.2 μm. All other simulation parameters (e.g., material model, cohesive zone model and analysis steps) are identical to those of the 2D model. Note that in the 3D model both tangential directions of the cohesive interface between the indenter and substrate are active. Therefore, the maximum stress criterion for damage initiation in Eq. (S4) is extended to the following for the 3D model

$$\max\left\{\langle\sigma_{\mathrm{n}}\rangle, \sigma_{\mathrm{s}}, \sigma_{\mathrm{t}}\right\} = \sigma_{\max} \quad \text{(S5)}$$

where σ_{n} , σ_{s} and σ_{t} represent the normal and the two tangential traction components, respectively.

Supplementary Figure 4b-4d compare results of the 2D and 3D models with indentation depth = $0.4R$ (i.e., 3.4 μm), velocity = 1 μm s^{-1}, and W_{ad} = 20 mJ m^{-2}. Creasing is observed in both 2D and 3D simulation results. Unlike the 2D model where the crease is straight along the out-of-plane direction (Supplementary Figure 4b), in the 3D model the crease is curved: it follows the circular contact perimeter between the indenter and the substrate at the leading edge and extends towards the side of the contact region, consistent with the experimental observation (Fig. 1c of the main text). Nevertheless, the crease shape on the central vertical cross-section of the 3D model is similar to that of the 2D model. In addition, the 2D and 3D models give a similar trend of lateral force (Supplementary Figure 4d). Recall that the lateral force in the 2D model is a line force (i.e., force per unit length along the out-of-plane direction). To enable comparison with the 3D model, we have converted the 2D line force to the total lateral force by multiplying the line force by the indenter diameter (17 μm). After conversion, we find the peak lateral forces in 3D and 2D models

are close. However, the 2D model predicts a higher compliance than the 3D model, i.e., it takes a longer lateral displacement to reach the peak lateral force in the 2D model. We attribute this difference to 3D effects of the crease morphology. Note that the formation of the crease increases the compliance for lateral motion of the indenter. In the 3D model, the crease size is the largest at the central cross-section and decreases towards the two sides. In contrast, in the 2D model the crease size is uniformly large along the out-of-plane direction, thereby leading to a larger increase in the compliance. Despite the difference in compliance, the comparison in Supplementary Figure 4 shows that the 2D model can capture the essential physics of crease formation. The 3D model contains 20~30 times more elements than the 2D model with the same mesh density, and thus is much more computationally expensive. Therefore, we adopted the 2D model for the parametric study on crease formation.

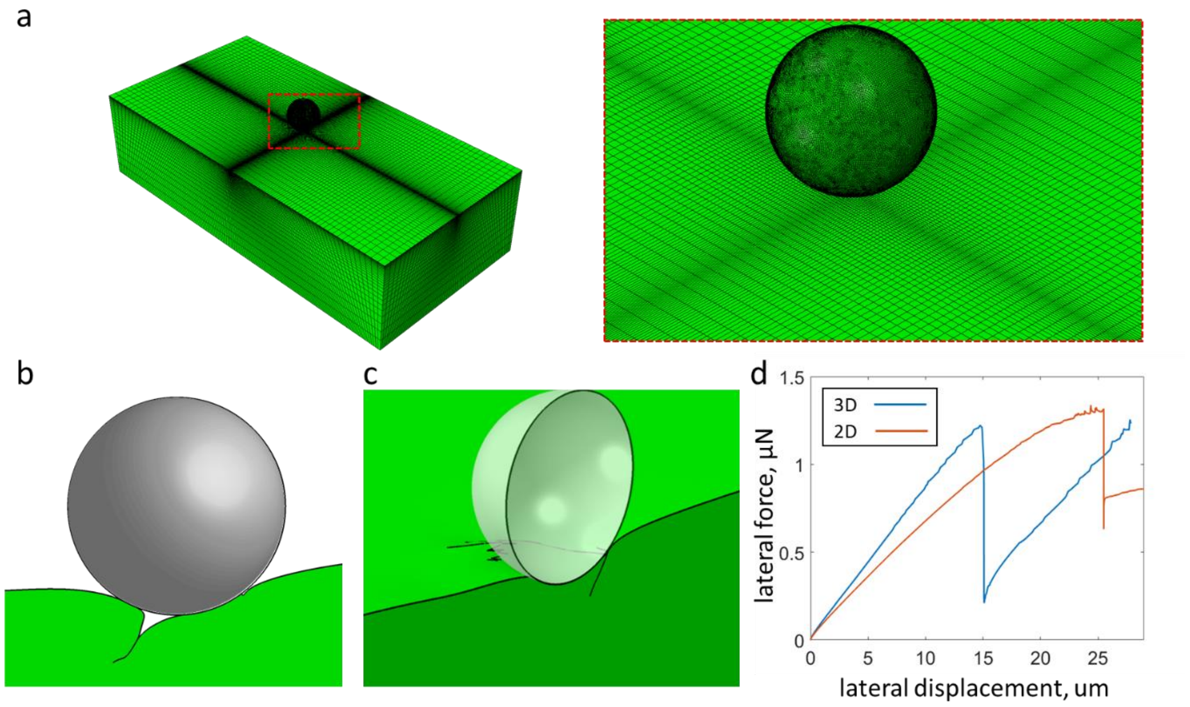

Supplementary Figure 4. Comparison between the 3D and 2D model under indentation depth = $0.4R$, lateral motion velocity = 1 μm s^{-1}, and W_{ad} = 20 mJ m^{-2}. (a) Geometry and mesh of the 3D model (left) and a zoomed-in view of the region near the indenter (right). (b) Snapshot of the 2D model at the peak lateral force. (c) Snapshot of the 3D model at the peak lateral force. (d) Lateral force versus the lateral displacement. For the 2D model, the line force is multiplied by the indenter diameter to enable comparison with the lateral force obtained from the 3D model.

Supplementary Note 4. Determination of the maximum compressive strain

In Fig. 4d of the main text, the maximum compressive strain in the substrate is shown to modulate crease nucleation. Here we illustrate how it is determined from simulation results. In a typical simulation, the maximum compressive normal strain along the horizontal direction at each time increment was extracted as the "spatial maximum compressive strain". Note that there are multiple strain measures in the continuum mechanics theory of finite deformation. We adopted the nominal strain (ABAQUS Analysis User's Guide 1.2.2) to define the "spatial maximum compressive strain" so that it can be compared with the critical creasing strain in the literature for global uniaxial compression.[6] Also, the "spatial maximum compressive strain" was sought over all the elements of the substrate but was found to occur at the substrate surface before the nucleation of creases. Supplementary Figure 5a shows the "spatial maximum compressive strain" as a function of the lateral displacement applied to the indenter. We emphasize that each data point of "spatial maximum compressive strain" was extracted as the maximum value from a strain field at a given time. For example, Supplementary Figure 5b shows the strain fields corresponding to the four points in Supplementary Figure 5a. Note that the logarithmic strain (or true strain) component ε_{xx} is shown in Supplementary Figure 5b for consistency since the strain fields are shown in the deformed configuration. We can see that the "spatial maximum compressive strain" reaches the peak value right before crease formation (i.e., snapshot iii) and then drops after crease formation. This peak value was extracted as the overall, both spatially and temporally, maximum compressive strain in Fig. 4d.

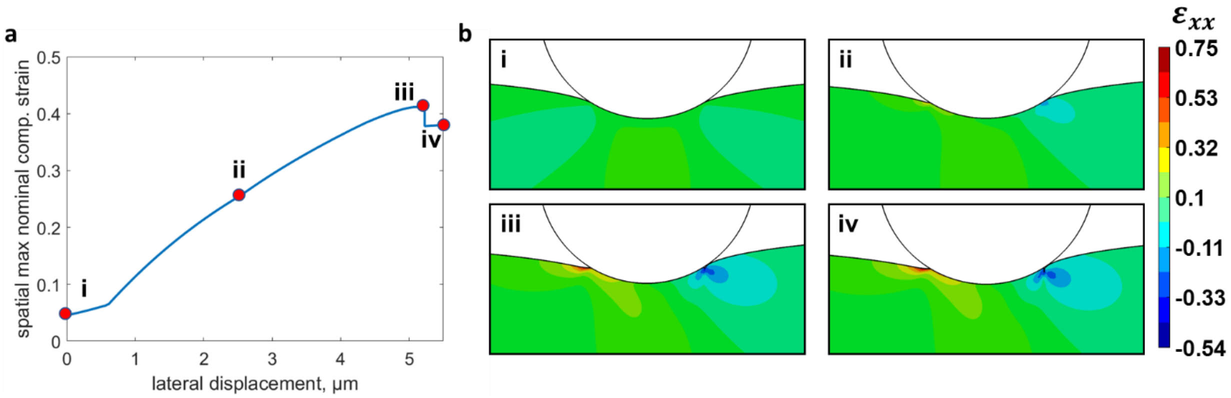

Supplementary Figure 5. Simulations of strain fields. (a) Representative result of the spatial maximum nominal compressive strain as a function of the lateral displacement applied to the indenter. The simulation was conducted for elastic substrate with $W_{\mathrm{ad}} = 8\ \mathrm{mJ/m^2}$ and $\sigma_{\max} = 8\ \mathrm{kPa}$ and no self-adhesion was considered. (b) Simulation snapshots of true strain field $\varepsilon_{\mathrm{xx}}$, the moments of which the snapshots were taken are marked correspondingly in Supplementary Fig. 5a.

Supplementary Note 5: Modeling self-adhesion within the crease

Self-adhesion between the contacting substrate surfaces within a crease was accounted for by adding another cohesive zone model for the self-contact of folded substrate surface. The cohesive zone model for self-adhesion also features a bilinear traction-separation relation (main text Fig. 4b). Two different self-adhesion conditions were studied in FEA, i.e., "sticky" self-adhesion and "slippery" self-adhesion, as defined below.

"Sticky" self-adhesion: for this condition, we adopted an isotropic traction-separation law for self-adhesion. This implies the cohesive parameters of self-adhesion, i.e., the initial stiffness K_{self}, strength $\sigma_{\text{max,self}}$, maximum separation $\delta_{\text{f,self}}$ and adhesion energy, $W_{\text{ad,self}}$ ($= \sigma_{\text{max,self}}\delta_{\text{f,self}}/2$), are the same along the normal and tangential directions. The maximum stress criterion was used for damage initiation, which now has the form:

$$\max\left\{\langle\sigma_{\text{n}}\rangle, \sigma_{\text{s}}\right\} = \sigma_{\text{max,self}} \tag{S6}$$

Specifically, the simulation results presented in main text Fig.5b were obtained using $W_{\text{ad,self}} = 20\ \text{mJ m}^{-2}$, which is approximately the surface tension of PDMS, $K_{\text{self}} = 2 \times 10^{11}\ \text{N m}^{-3}$, $\sigma_{\text{max,self}} = 20$ kPa and $\delta_{\text{f,self}} = 2$ μm.

"Slippery" self-adhesion: to enable slip within the crease while retaining self-adhesion, we implemented different cohesive parameters for the normal and tangential directions of the crease:

- In the normal direction (i.e., perpendicular to the crease), we used the same cohesive parameters as those of "sticky" self-adhesion, i.e., $W_{\text{ad,self}} = 20\ \text{mJ m}^{-2}$, $K_{\text{self,n}} = 2 \times 10^{11}\ \text{N m}^{-3}$, $\sigma_{\text{max,self,n}} = 20$ kPa and $\delta_{\text{f,self,n}} = 2$ μm. Here the subscript n denotes the normal direction.

- In the tangential directions (i.e., parallel to the crease and denoted with subscripts s and t), the cohesive parameters are: $K_{\text{self,s}} = 4 \times 10^{4}\ \text{N m}^{-3}$, $\sigma_{\text{max,self,s}} = 0.01$ kPa and $\delta_{\text{f,self,s}} = 4$ mm (same parameters for the other tangential direction t). Although these parameters correspond to the same adhesion energy as the normal direction, i.e., $W_{\text{ad,self}} = 20\ \text{mJ m}^{-2}$, the ultra-low $K_{\text{self,s}}$ and $\sigma_{\text{max,self,s}}$ allow the relative separation along the tangential

directions of self-contact to be large, thereby effectively enabling slippage within the crease. Specifically, the maximum tangential separation $\delta_{\mathrm{f,self,s}}$ was set to be 4 mm, which is several orders of magnitude larger than the indenter radius (i.e., 8.5 μm). This ensures that $\delta_{\mathrm{f,self,s}}$ is never achieved in our simulations and thus only normal separation can eventually cause interface damage of self-adhesion. Because of the anisotropic cohesive parameters along normal and tangential direction, the maximum stress criterion for damage initiation now reads as:

$$\max\left\{\frac{\langle\sigma_{\mathrm{n}}\rangle}{\sigma_{\mathrm{max,self,n}}},\frac{\sigma_{\mathrm{s}}}{\sigma_{\mathrm{max,self,s}}}\right\}=1. \tag{S7}$$

Supplementary Note 6. Capturing the trend of peak lateral force in simulations

We use the 2D FEA model with full visco-hyperelastic substrate and "slippery" self-adhesion (Supplementary Note 5) to simulate the crease morphology and peak lateral force under different lateral velocity v and relative depth d/R. To capture both creasing and non-creasing cases, we set the interfacial strength between the indenter and substrate, $\sigma_{\max}$, to be 20 kPa (creasing) or 2 kPa (non-creasing) while keeping the final separation $\delta_{\mathrm{f}} = 2\ \mu\mathrm{m}$. Correspondingly, the adhesion energy W_{ad} is 20 mJ m^{-2} (creasing) or 2 mJ m^{-2} (non-creasing). The simulation results, summarized in Supplementary Figure 6, demonstrate qualitative agreement with the experimental results in Fig. 2 of the main text. Specifically, in case of creasing (Supplementary Figure 6a), the crease does not open until it progresses through the contact zone and reaches the trailing edge where a region of local tension exists. The non-creasing case (Supplementary Figure 6b) features a smooth build up with substrate deformation with smaller compressive strain along the horizontal direction than the creasing case. The lateral force versus lateral displacement curve (Supplementary Figure 6c) and the peak lateral force under increasing depth and lateral velocity (Supplementary Figure 6d) show similar trends as those in Fig. 2c and 2d of the main text. Recall that the lateral force obtained from simulations is a line force (i.e., force per unit length along the out-of-plane direction) because of the 2D plane strain geometry of FEA model.

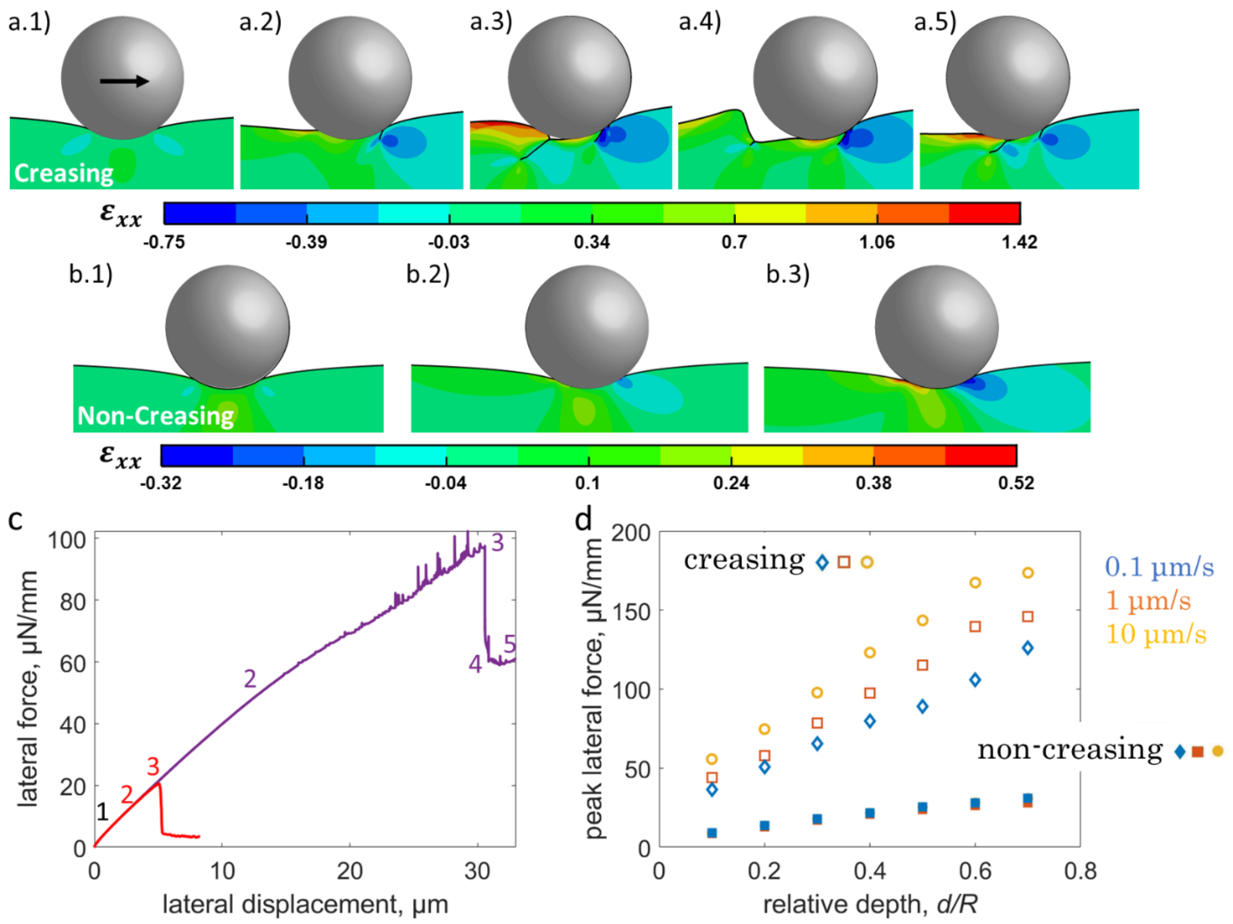

Supplementary Figure 6. Capturing the creasing and non-creasing cases in FEA simulations. a) Representative simulation results for creasing case obtained using lateral velocity v =1 μm s^{-1}, relative depth d/R=0.4, and interfacial strength $\sigma_{\max}$ = 20 kPa . The crease morphology resembles those observed experimentally in Fig. 2a of main text. a.1) The microsphere in contact with the surface prior to lateral motion. a.2) Initiation of creasing. a.3) The moment before the first crease releases, which is at F_{peak}. a.4) The moment after the first crease releases from the back edge and the lateral force drops. a.5) The moment at which the second crease progresses to the center of contact region. b) Representative simulation results for non-creasing case obtained using v = 1 μm s^{-1}, d/R=0.4, and $\sigma_{\max}$ = 2 kPa . b.1) The microsphere in contact with the surface prior to lateral motion. b.2) The contact shape about midway to F_{peak}. b.3) The contact shape at the peak lateral force F_{peak}. c) Lateral force versus lateral displacement curves for creasing and non-creasing simulations; the numbered points correspond to the snapshots in parts a and b. d) Simulation results of F_{peak} for creasing and non-creasing cases as a function of d/R and v.

Supplementary Note 7. Fluorescence intensity with crosslinking and potential top layer effects

In this supplementary note, we expand on the question of fluorescence intensity and potential top layer effects. As described in the discussion of the main text, we observed a change in the fluorescent intensity of the crosslinkable, fluorescein diacrylate dye before and after curing (Supplementary Figure 7). This suggests that a bright line at the PDMS surface may be the result of additional crosslinking (e.g. Fig. 2a in the main text). Additionally, UVO is known to create a glassy layer at the surface of PDMS. However, at our short times (tens of seconds), it is likely negligible in terms of the mechanics of creasing (a UVO layer is likely less than ~1 nm).

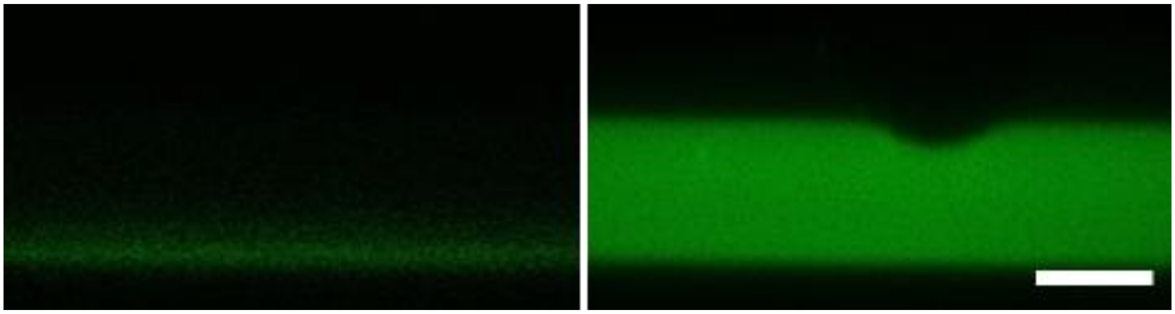

Supplementary Figure 7. Fluorescent signal of the Fluorescein diacrylate dye molecule before (left) and after curing (right). Note that the images are taken with the same microscope parameters (e.g. excitation laser power and collection wavelength). Scale bar: 20 μm.

To confirm that a potential stiff layer does not govern crease formation, we ran simulations with the 2D model to study the effect of stiff layer. In these simulations, the substrate was modelled as an incompressible neo-Hookean solid with a shear modulus of 1.89 kPa (i.e., neglecting viscoelasticity). Self-adhesion within the crease was also neglected for simplicity. We added a thin layer which is also modelled as an incompressible neo-Hookean solid but with a shear modulus that is 100 times larger than the substrate. The thickness of this stiff layer was varied from 50 nm up to 400 nm. The stiff layer was meshed by squared elements with a size less than 200 nm (recall that the smallest element size in the substrate was also 200 nm). Specifically, for 400 nm-thick layer, the mesh size in the layer was 200 nm × 200 nm. For 50 nm-thick layer, we refined the mesh such that the mesh size in the layer was 50 nm × 50 nm. To improve accuracy, the element type in these simulations were changed to CPE4H. The simulation results on the

effect of the stiff thin layer are summarized in Supplementary Figure 8. Specifically, Supplementary Figure 8a plots the minimum interfacial strength σ_{max} required for crease formation as a function of the thin layer thickness, and Supplementary Figure 8b shows the peak lateral forces corresponding to the data in Supplementary Figure 8a. Interestingly, when the stiff layer becomes thicker, a higher interfacial strength is required for crease formation. These results are consistent with the expectation that the stiff layer is associated with a bending stiffness, which induces an additional elastic energy penalty to surface creasing. Consequently, higher compressive strain is required for crease formation with a thicker stiff layer; the peak lateral force also increases with thickness correspondingly. Hence, it is unlikely that a ~1 nm layer in our experiments, created by short UVO exposure, will play a significant role in crease formation.

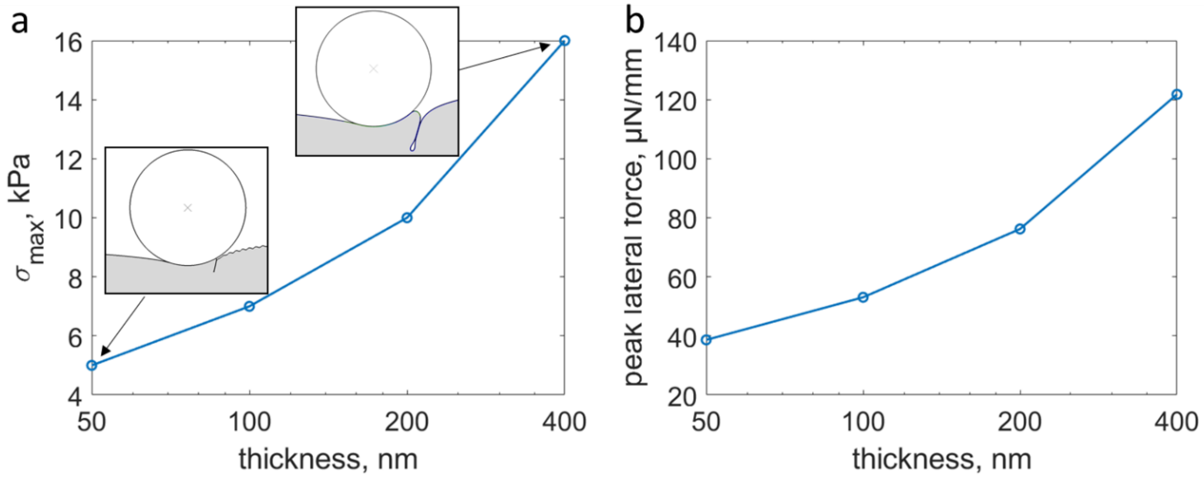

Supplementary Figure 8. Simulating effect of the stiff layer on an elastic substrate with indentation depth = 0.4*R*. (a) Minimum interfacial strength required for crease formation as a function of the stiff layer thickness. The insets are the simulation snapshots at peak lateral force for the two cases with layer thickness being 50 nm or 400 nm. The other cohesive parameters are the same as those described in Supplementary Note 2 (i.e., $\delta_{\text{f}} = 2$ μm and $K = 2 \times 10^{11}$ N m^{-3}). (b) Peak lateral force corresponding to the data in part (a).

References:

1 Simo, J. C. On a fully three-dimensional finite-strain viscoelastic damage model: formulation and computational aspects. *Computer methods in applied mechanics and engineering* **60**, 153-173 (1987).

2 Fernanda, M., Costa, P. & Ribeiro, C. Parameter estimation of viscoelastic materials: a test case with different optimization strategies. *AIP Conference Proceedings* **1389**, 771-774 (2011).

3 Glover, J. D. & Pham, J. T. Capillary-driven indentation of a microparticle into a soft, oil-coated substrate. *Soft Matter* **16**, 5812-5818 (2020).

4 Hui, C., Ruina, A., Long, R. & Jagota, A. Cohesive zone models and fracture. *The Journal of Adhesion* **87**, 1-52 (2011).

5 Papangelo, A. & Ciavarella, M. On mixed-mode fracture mechanics models for contact area reduction under shear load in soft materials. *Journal of the Mechanics and Physics of Solids* **124**, 159-171 (2019).

6 Hong, W., Zhao, X. & Suo, Z. Formation of creases on the surfaces of elastomers and gels. *Applied Physics Letters* **95**, 111901 (2009).
