# Supplementary material for: Creasing in microscale, soft static friction
Source: Nat Commun. 2023 Apr 24;14:2362. doi: 10.1038/s41467-023-38091-7 (PMC10126204; doi:10.1038/s41467-023-38091-7)
Supplement: Supplementary file 3 — Description of Additional Supplementary Files [file 41467_2023_38091_MOESM3_ESM.pdf]

## Description of Additional Supplementary Files

**Supplementary Movie 1:** A video showing the creasing case. The creasing force data from this video is plotted in Figure 2c of the main text. Lateral motion started at ~18 s. The substrate is being pulled to the left.

**Supplementary Movie 2:** A video showing the non-creasing case. The non-creasing data from this video is plotted in Figure 2c of the main text. Lateral motion started at ~45 s. The substrate is being pulled to the left.

**Supplementary Movie 3:** A video showing the non-creasing case in reflection mode. Lateral motion started at ~15 s. The substrate is being pulled to the left.

**Supplementary Movie 4:** A video showing the creasing case in reflection mode. Lateral motion started at ~33 s. The substrate is being pulled to the left. Though the crease is difficult to visualize in reflection, a small dip down of the surface can be seen at the front of the probe, which is more similar in geometry to the creasing case than the non-creasing case. Additionally, the release at the back of the probe appears larger. These observations suggest that crease formation is not due to the presence of dye in the sample. Moreover, it suggests that creasing is not caused by the dye crosslinking to the network due to light exposure.
